# Increased Risk of Site-Specific Cancer in People with Type 2 Diabetes: A National Cohort Study

**DOI:** 10.3390/ijerph17010246

**Published:** 2019-12-30

**Authors:** Donata Linkeviciute-Ulinskiene, Ausvydas Patasius, Lina Zabuliene, Rimantas Stukas, Giedre Smailyte

**Affiliations:** 1 Institute of Biomedical Sciences, Department of Pathology, Forensic Medicine and Pharmacology, Faculty of Medicine, Vilnius University, Ciurlionio g. 21, 03101 Vilnius, Lithuania; 2 Laboratory of Cancer Epidemiology, National Cancer Institute, P. Baublio g. 3b, 08406 Vilnius, Lithuania; ausvydas.patasius@nvi.lt (A.P.); giedre.smailyte@nvi.lt (G.S.); 3 Institute of Health Sciences, Faculty of Medicine, Vilnius University, Ciurlionio g. 21, 03101 Vilnius, Lithuania; rimantas.stukas@mf.vu.lt; 4 Institute of Clinical Medicine, Faculty of Medicine, Vilnius University, Santariskiu g. 2, 08406 Vilnius, Lithuania; lina.zabuliene@mf.vu.lt

**Keywords:** diabetes, cancer, risk, cohort, population study

## Abstract

A retrospective cohort design was used with the objective to evaluate cancer risk among people with type 2 diabetes mellitus (T2DM) in Lithuania. The cohort was established by identifying all patients with the first diagnosis of T2DM in the National Health Insurance Fund database during 2000–2012. Cancer cases were identified by record linkage with the Lithuanian Cancer Registry. Standardized incidence ratios (SIRs) were calculated. Of the 127,290 people that were included, 5959 cases of cancer in men and 6661 cancer cases in women with T2DM were observed. A statistically significant increase in risk for all cancer sites was observed in women, SIR 1.16 (95% CI 1.14–1.19), but not in men, SIR 1.00 (95% CI 0.98–1.03). Among males, a significant increase of liver (SIR 2.11, 95% CI 1.79–2.49]), pancreas (SIR 1.77, 95% CI 1.57–1.99), kidney (SIR 1.46 95% CI 1.31–1.62), thyroid (SIR 1.83, 95% CI 1.32–2.54), colorectal (SIR 1.23, 95% CI 1.14–1.31]), skin melanoma (SIR 1.40, 95% CI 1.11–1.76), and non–melanoma skin (SIR 1.14, 95% CI 1.05–1.23) cancer was observed. For females with T2DM, a significant increase in risk of cancer of the liver (SIR 1.45, 95% CI 1.17–1.79), pancreas (SIR 1.74, 95% CI 1.56–1.93), kidney (SIR = 1.43, 95% CI 1.28–1.60), thyroid (SIR = 1.40, 95% CI 1.22–1.62), breast (SIR = 1.24, 95% CI 1.17–1.31), and corpus uteri (SIR 2.07, 95% CI 1.93–2.21) was observed. In conclusion, people with T2DM in Lithuania had an increased risk of site-specific cancer.

## 1. Introduction

Diabetes mellitus and cancer are complex and growing worldwide health problems, associated with severe acute and chronic complications affecting both the quality of life and survival. According to the International Diabetes Federation, it is estimated that there were 425 million people with diabetes in 2017, and by 2045 the number will rise to 629 million, increasing by 48% [1]. Whereas cancer is the second leading cause of death globally, and is responsible for an estimated 9.6 million deaths in 2018, which means that about one in six deaths is due to cancer [2]. Furthermore, about 8–18% of cancer patients also have diabetes [3]. Since the incidence of cancer and diabetes is predicted to increase in the following decades, this emphasizes the relevance of adequate prevention, early detection, and appropriate management of both diseases worldwide.

A wide range of epidemiological studies on cancer and diabetes interactions have been conducted and diabetes (primarily T2DM) was shown to be associated with increased risk for some cancer sites (e.g., liver, pancreas, endometrium, colon and rectum, breast, bladder), for other sites there appears to be no association or the evidence is inconclusive [4]. There are several explanations of the observed associations between T2DM and cancer. First of all, a causal effect of hyperglycaemia, hyperinsulinemia, and inflammation can explain heightened risk of cancer of many sites, in addition to confounding from common risk factors such as adiposity, age, and physical activity. Reverse causality is another potential alternative hypothesis for the associations observed, such as in T2DM and risk of developing hepatocellular and pancreatic cancer, because these cancers can lead to dysfunction of insulin secretion, glucose metabolism, and gluconeogenesis [4]. Evidence suggests that even when these factors are taken into account, observed associations between cancer and diabetes remain [5]. Ascertainment bias might also exist since there is heightened medical investigation of diabetic patients, particularly with a new diagnosis of diabetes, but studies have still found significant associations even after the exclusion of the first few years of follow-up after diagnosis [6]. Additionally, it has been considered that, since T2DM is typically an underdiagnosed disease, and cancer is more frequent in patients with diabetes, the association between these diseases might be undervalued [4].

Neither diabetes, nor cancer patients can be considered a very homogeneous cohort, with great differences in the disease course, not to mention risk factors, medication for the treatment of the diseases itself and comorbidities, nutritional characteristics, nationality, ethnicity, etc., which may impact the course of diabetes and the risk of cancer and provide a basis for study bias. Therefore, this association of the two diseases remains a question that is relevant now and in years to come. 

Most of the previous studies have focused on specific cancer sites. This study provides detailed evaluation of cancer risk by cancer site in the diabetic patient cohort. The aim of this study was to describe the cancer risk pattern among T2DM patients in the whole population of Lithuania. 

## 2. Research Design and Methods

### 2.1. Study Design and Data Source

A retrospective cohort study was conducted to examine the relationship between T2DM and site-specific cancer risk. Due to the design of the study, informed consent of individuals was not obtained. The study was approved by the Vilnius Regional Biomedical Research Ethics Committee.

Lithuania has a compulsory health insurance system, therefore, the vast majority of healthcare services are covered by the National Health Insurance Fund (NHIF). NHIF database was created in 1999 for the management, storage, analysis and reporting of the services provided by healthcare organizations. The database includes demographic data and entries on the primary, secondary and tertiary healthcare services provided, hospital admissions, and prescriptions of reimbursed medicine.

The cohort was established by identifying all male and female patients with the first entry of T2DM diagnosis (International Classification of Diseases Australian modification, ICD-10-AM code E11) in the NHIF database from 1 January 2000, until 31 December 2012. To minimize the risk of false type 2 diabetes classification, T2DM status was assigned to patients who were reported as T2DM patients and received prescriptions of antidiabetic medications in the NHIF database. 

To ascertain cancer incidence in the cohort, diabetes records were linked to the Lithuanian National Cancer Registry by use of a personal identification number assigned to all Lithuanian citizens, using data up to and including 31 December 2012. The Cancer Registry is a nationwide population-based cancer registry that contains personal and demographic information on all people diagnosed with cancer in Lithuania since 1978. It is a statutory requirement to notify the registry of all cases of malignant neoplasms. All recorded cancers in the Cancer Registry are coded according to the ICD-10-AM.

Patients with T2DM aged 40 years or older were included into the final cohort. We excluded 8230 cases with a cancer diagnosis before the diagnosis of diabetes. The cohort exit date was defined as either the date of death, emigration or 31 December 2012, whichever came first. Overall, 127,290 people (78,823 women and 48,467 men) were included into the study, with the observation period of 828,242.3 person years.

### 2.2. Statistical Analysis

We calculated standardized incidence ratios (SIRs) for site-specific and overall cancer as a ratio of observed number of cancer cases in people with T2DM to the expected number of cancer cases in the underlying general Lithuanian population. 

Expected number of cancer cases were calculated by multiplication of the exact person-years under observation in the cohort by sex, calendar year, and 5 year age-group specific national incidence rates [7]. The person-time of observation was computed from the date of the first entry of T2DM diagnosis in the NHIF database until the cohort exit date. 95% confidence intervals for the SIRs were estimated assuming the number of observed cases follows Poisson distribution. 

All statistical analyses were carried out using STATA 11 statistical software (StataCorp. 2009. Stata Statistical Software: Release 11.0. College Station, TX, USA). The study was conducted in accordance with the Declaration of Helsinki, and the protocol was approved by the Vilnius Regional Biomedical Research Ethics Committee (Nr. 158200-17-913-423).

## 3. Results

During 12 years of follow-up, 5959 cases of cancer in men and 6661 cases of cancer in women with T2DM were diagnosed. The study population’s baseline characteristics are described in Table 1. Statistically significant increase in risk for overall cancer was observed in women, SIR 1.16 (95% CI 1.14–1.19), but not in men, SIR 1.00 (95% CI 0.98–1.03) (Table 2).

Among males with T2DM, significantly increased risk was found for cancer of the liver (SIR 2.11, 95% CI 1.79–2.49), pancreas (SIR 1.77, 95% CI 1.57–1.99), kidney (SIR 1.46, 95% CI 1.31–1.62), and thyroid (SIR 1.83, 95% CI 1.32–2.54). Colorectal cancer (SIR 1.23, 95% CI 1.14–1.33), skin melanoma (SIR 1.40, 95% CI 1.11–1.76), non–melanoma skin cancer (SIR 1.14, 95% CI 1.05–1.23), male genital organ (SIR 1.86, 95% CI 1.27–2.71), and other endocrine organ (SIR 1.96, 95% CI 1.05–3.64) cancer risk was elevated significantly as well.

For females with T2DM, similarly to males, significantly increased risk was found for cancer of the liver (SIR 1.45, 95% CI 1.17–1.79), pancreas (SIR 1.74, 95% CI 1.56–1.93), kidney (SIR 1.43, 95% CI 1.28–1.60), and thyroid (SIR 1.40, 95% CI 1.22–1.62). Breast (SIR 1.24, 95% CI 1.17–1.31) and corpus uteri (SIR 2.07, 95% CI 1.93–2.21) cancer risk was significantly increased as well. 

There was also an inverse association with several cancer sites. A decreased incidence in cancer of the mouth (SIR = 0.49, 95% CI = 0.39-0.62), oesophagus (SIR 0.50, 95% CI 0.37–0.66), larynx (SIR 0.57, 95% CI 0.44-0.73), lung and trachea (SIR 0.53, 95% CI 0.48–0.58) in men, and multiple myeloma (SIR 0.74, 95% CI 0.57-0.96) and leukemia (SIR 0.81, 95% CI 0.68–0.97) in women was observed (Table 2).

## 4. Discussion

Results of this large population-based retrospective study based on medical service records and the data of the Cancer Registry showed significant increase in site-specific cancer risk among T2DM patients. We found different risk estimates for overall cancer among males and females. The male vs. female cancer risk difference observed in the patient cohort can be partly explained by an increased number of the most common sex-specific, corpus uteri and breast, cancer in the female cohort group. When these cancers are excluded from the calculation for overall cancer risk, a smaller, yet statistically significant increase for overall cancer in women remains (SIR 1.06, 95% CI 1.03–1.09). Men, on the other hand, had an increased risk for more types of cancer than women, however, these cancers where not so common and did not affect the overall cancer risk.

SIR estimates for overall cancer risk in T2DM patients obtained in our study are comparable to previously performed European cohort studies. A cohort study in Denmark showed a 10% increase in overall cancer for both men and women, with highest risk of hepatic, pancreatic, kidney, and corpus uteri cancer [8]. A cohort study from Tyrol, Austria revealed a neutral risk for overall cancer, with significant increase in cancer of the pancreas and corpus uteri for women, and cancer of the liver and pancreas for men. [9]. A recent Finish cohort study showed an 18% higher risk of cancer for diabetic females and 14% for diabetic males, with the liver and pancreas being the sites at highest risk among both sexes [10]. 

According to a recent meta-analysis of 121 cohorts (19,239,302 individuals; 1,082,592 events) on all-site cancer risk, the risk of cancer among patients with diabetes is shown to be higher: the pooled adjusted relative risk (RR) for all-site cancer associated with diabetes was 1.27 (95% CI 1.21–1.32) in women and 1.19 (95% CI 1.13–1.25) in men [11]. 

With regards to SIR estimates on site-specific cancer incidence, our results are comparable to previous meta-analyses [12,13,14,15,16,17,18,19,20,21,22]. We found a twofold rise in liver cancer incidence among men with T2DM and a 45% rise among women with T2DM compared with the general population. These findings have been similar to those observed in the meta-analysis by Wang et al., 2012, where the combined risk estimate for hepatocellular cancer was 2.31 (95% CI 1.87–2.84) among diabetic individuals [12]. A more recent meta-analysis of cohort studies on T2DM and gender differences in liver cancer by Wang et al., 2016, associated T2DM with an elevated liver cancer incidence in both men (summary RR 2.16, 95% CI 1.74–2.69) and women (summary RR 1.85, 95% CI 1.40–2.44). In addition, the study showed that T2DM and liver cancer association is confounded by smoking and body mass index in both men and women. Results revealed a significantly stronger T2DM and liver cancer association in non-Asian than in Asian women and men [13]. 

We observed a more than 75% higher risk of pancreatic cancer in both men and women with T2DM. A meta-analysis by Song et al., 2015, found that long diabetes duration was associated with a 1.5- to 1.7-fold increased risk of pancreatic cancer [14]. Although reverse causality has been suspected, constant elevated risk of pancreatic cancer after longer periods of follow-up demonstrate that this is doubtful [6,14]. 

We found a 40% increased risk of kidney cancer in both women and men. This finding is similar to a meta-analysis by Bao et al., 2013, which showed a slightly stronger positive relation in women (RR 1.47, 95% CI 1.18–1.83) than in men (RR 1.28, 95% CI 1.10–1.48) [15]. Analyses indicate that the increased risk of kidney cancer is independent of alcohol consumption, BMI, obesity, and smoking [15]. 

In our study, women with T2DM had a 40% and men had an 80% higher risk for thyroid cancer, although it remained a rare form of cancer in men. The results on thyroid cancer are inconsistent among studies. Our results for women are comparable with a 2014 meta-analysis by Yeo et al., which showed a 30% increased risk in women, but not in men. The risk was higher among women living in areas where thyroid cancer is more common relative to other geographic areas [16]. However, a later prospective study by Luo et al. [23] performed in the US of 147 thousand postmenopausal women showed no significant association between diabetes and thyroid cancer, even when diabetes treatment and duration was taken into account [23]. We hypothesize that the higher estimates for thyroid cancer, especially among men, similarly to increasing rates of thyroid cancer in the whole population, can be explained by the fact that diabetic patients in Lithuania usually undergo thyroid ultrasound scanning routinely, and ultrasound guided fine needle biopsy if needed, therefore even micro-carcinomas can be detected early [24]. 

Our study showed a 23% increased colorectal cancer risk in men. Several meta-analyses of cohort and case-control studies demonstrated an increased risk of colorectal cancer in both women and men with diabetes [17,18,19]. A latest meta-analysis of 38 cohort studies suggested that there was no evidence of a sex difference for colorectal cancer among patients with diabetes compared to those without diabetes (ratio of RR 0.99, 95% CI 0.94–1.04) [18]. T2DM and colon cancer share common risk factors such as obesity and smoking, but even when adjusted for these factors, the positive association remains [19].

Some significant associations were sex-specific. Women with diabetes had a two-fold increased risk of corpus uteri cancer, which is a well-recognized association and our results are comparable to the latest meta-analyses [20,21]. Our study showed a significantly higher risk for breast cancer, which is comparable with a meta-analysis by Liao et al., 2011, according to which, the association between diabetes and breast cancer was the most obvious in Europe (RR 1.88, 95% CI 1.56–2.25), followed by America (RR 1.16, 95% CI 1.12–1.20), but it was not significant in Asia (RR 1.01, 95% CI 0.84–1.21) [22].

There was no reduction in prostate cancer risk among men with T2DM in our study, in contrast to a recent meta-analysis, which showed a decreased risk of prostate cancer in diabetic men (RR 0.86, 95% CI 0.80–0.92) [25]. In Lithuania an official nationwide prostate cancer early detection program was introduced in 2006, resulting in subsequent prostate cancer incidence peaks [26]. Therefore, obtained risk excess of prostate cancer in our study group could in part be explained by surveillance bias since patients with diabetes are under increased surveillance and are more likely to undergo additional medical examinations including prostate-specific antigen (PSA) testing. The use of PSA testing as an early detection tool may cause overdiagnosis or detection of indolent tumors [27]. 

We found a lower risk of lung, tracheal, oesophageal, mouth, and laryngeal cancer in diabetic men. In comparison, a meta-analysis of 13 studies indicates that DM is positively correlated with oesophageal cancer, although there were limitations on potential clinical confounding factors in each study included in the meta-analysis [28]. Cancers of the oral cavity, pharynx, oesophagus, and larynx, together with cancers of the trachea and lungs, are commonly associated with smoking and alcohol use [29]. Lower risk of some cancers in our study may be explained by a different distribution of well-known risk factors in diabetic patients compared to the general population.

Our study has strengths and limitations. The strengths of this study are the evaluation based on real life data from two well established registries. This study is population based with a large sample size, long follow-up time, and the diagnosis of T2DM is well defined. We add more precise risk estimates for some of the less common cancers to the current literature on T2DM and cancer incidence. 

The limitations of our study are those typical in observational studies, such as lack of confounding factors and the potential interactions of diabetes and cancer, for example, problems involving reverse causality, common risk factors and competing risks. It is possible that we have underestimated the impact since standard for calculation of SIR was given by the whole population, which may also include undiagnosed T2DM patients or those treated outside the national health insurance scheme. 

## 5. Conclusions

In conclusion, our study shows a heightened risk of overall cancer in women and several types of site-specific cancer in patients with T2DM in Lithuania. Significantly increased risk was found for cancers of the liver, pancreas, kidney and thyroid in both men and women. Risk of colorectal cancer, skin melanoma, and non–melanoma skin cancer was elevated among males, and risk of breast and corpus uteri cancers among females.

## Figures and Tables

**Table 1:** Characteristics of the study population.

Characteristic	Males (48,467)	Females (78,823)	Total (127,290)
Mean age at study entry, y (SD)	59.7 (10.4)	64.0 (10.6)	62.4 (10.7)
40–49 y, No. (%)	9639 (20)	7971 (10)	17,610 (14)
50–59 y, No. (%)	15,808 (32)	20,617 (26)	36,425 (29)
60–69 y, No. (%)	14,371 (30)	26,254 (33)	40,625 (32)
70+	8649 (18)	23,981 (31)	32,630 (25)
Mean duration of follow-up, y (SD)	6.1 (3.9)	6.8 (4.0)	6.5 (3.9)
Total No. of person-years at risk	295,390	532,852	828,242
No. of cancer cases	5959	6661	12620
Mean age at cancer diagnosis, y (SD)	69.0 (8.8)	70.4 (9.4)	69.7 (9.1)

SD, standard deviation.

**Table 2:** Observed and expected numbers of cancer cases and standardized incidence ratios with 95% confidence intervals among Lithuanian type 2 diabetic patients during 2000–2012 by site according to the International Classification of Diseases Australian Modification.

Primary Site	ICD 10-AM Code	Males				Females			
		Obs	Exp	SIR	95% CI	Obs	Exp	SIR	95% CI
All Sites	C00–C96	5959	5934	1.00	0.98–1.03	6661	5724	1.16	1.14–1.19
Lip	C00	21	23	0.93	0.61–1.43	9	11	0.79	0.41-1.52
Mouth and pharynx	C01–C14	73	149	0.49	0.39–0.62	32	40	0.81	0.57–1.14
Oesophagus	C15	46	93	0.50	0.37–0.66	16	20	0.79	0.49–1.29
Stomach	C16	325	339	0.96	0.86–1.07	303	305	0.99	0.89–1.11
Colon, rectum, rectosigmoid, anus	C19–C21	627	508	1.23	1.14–1.33	682	640	1.07	0.99–1.15
Liver	C22	139	66	2.11	1.79–2.49	86	59	1.45	1.17–1.79
Gallbladder, bile ducts	C23, C24	28	24	1.17	0.81–1.70	66	63	1.05	0.82–1.33
Pancreas	C25	265	150	1.77	1.57–1.99	325	187	1.74	1.5–61.93
Other digestive organs	C17, C26, C48	15	17	0.89	0.54–1.47	42	29	1.45	1.07–1.97
Nasal cavity, middle ear, accessory sinuses	C30, C31	7	10	0.68	0.32–1.42	6	8	0.76	0.34–1.69
Larynx	C32	63	111	0.57	0.44–0.73	6	7	0.83	0.37–1.85
Lung, trachea	C33, C34	436	828	0.53	0.48–0.58	183	211	0.87	0.75–1.00
Other respiratory tract	C37–C39	4	6	0.62	0.23–1.64	5	5	0.99	0.41–2.37
Bone and connective tissue	C40, C41, C45–C47, C49	28	29	0.98	0.68–1.42	33	36	0.91	0.65–1.28
Skin, melanoma	C43	72	52	1.40	1.11–1.76	101	111	0.91	0.75–1.10
Skin, non-melanoma	C44	566	498	1.14	1.05–1.23	1033	1049	0.98	0.93–1.05
Breast	C50	10	9	1.17	0.63–2.17	1114	900	1.24	1.17–1.31
Vulva	C51	-	-	-	-	53	45	1.17	0.89–1.53
Cervix uteri	C53	-	-	-	-	198	212	0.93	0.81–1.07
Corpus uteri	C54, C55	-	-	-	-	872	422	2.07	1.93–2.21
Ovary	C56	-	-	-	-	249	264	0.94	0.83–1.07
Other female genital organs	C52, C57–C58	-	-	-	-	13	17	0.76	0.44–1.31
Male genital organs	C60, C63	27	15	1.86	1.27–2.71	-	-	-	-
Prostate	C61	2164	2074	1.04	1.00–1.09	-	-	-	-
Testis	C62	4	4	1.04	0.39–2.78	-	-	-	-
Kidney	C64	336	231	1.46	1.31–1.62	312	218	1.43	1.28–1.60
Urinary bladder	C67	214	208	1.03	0.90–1.18	97	86	1.13	0.93–1.38
Other urinary tract	C65, C66, C68	10	10	0.99	0.53–1.84	13	11	1.19	0.69–2.05
Eye and adnexa	C69	8	7	1.13	0.56–2.26	12	13	0.95	0.54–1.68
Central nervous system	C70–C72	46	61	0.76	0.57–1.01	88	90	0.98	0.79–1.21
Thyroid	C73	36	20	1.83	1.32–2.54	196	140	1.40	1.22–1.62
Other endocrine organs	C74, C75	10	5	1.96	1.05–3.64	9	7	1.36	0.71–2.62
Other and ill-defined sites	C76–C80	138	130	1.06	0.90–1.25	173	147	1.18	1.01–1.37
Non-Hodgkin lymphoma	C81	5	6	0.78	0.32–1.87	7	8	0.84	0.40–1.77
Hodgkin lymphoma	C82–C85	72	82	0.87	0.69–1.10	145	130	1.12	0.95–1.31
Myeloma	C90	40	40	0.99	0.73–1.35	55	75	0.74	0.57–0.96
Leukaemia	C91–C95	123	127	0.97	0.81–1.16	126	155	0.81	0.68–0.97
Other and unspecified malignant neoplasms of lymphoid, haematopoietic and related tissue	C88, C96	1	4	0.26	0.04–1.87	1	4	0.25	0.04–1.80

Obs, observed; Exp, expected; SIR, standardized incidence ratios; CI, confidence interval; ICD10-AM, International Classification of Diseases Australian Modification.

## Abbreviations

T2DM	Type 2 diabetes mellitus
SIR	standardized incidence ratio
NHIF	National Health Insurance Fund
CI	confidence interval
RR	relative risk
